# Detection of pancreatic cancer with two- and three-dimensional radiomic analysis in a nationwide population-based real-world dataset

**DOI:** 10.1186/s12885-023-10536-8

**Published:** 2023-01-17

**Authors:** Dawei Chang, Po-Ting Chen, Pochuan Wang, Tinghui Wu, Andre Yanchen Yeh, Po-Chang Lee, Yi-Hui Sung, Kao-Lang Liu, Ming-Shiang Wu, Dong Yang, Holger Roth, Wei-Chih Liao, Weichung Wang

**Affiliations:** 1 Data Science Degree Program, National Taiwan University and Academia Sinica, Taipei, Taiwan; 2 Department of Medical Imaging, National Taiwan University Hospital, National Taiwan University College of Medicine, Taipei, Taiwan; 3 Department of Computer Science and Information Engineering, National Taiwan University, Taipei, Taiwan; 4 Institute of Applied Mathematical Sciences, National Taiwan University, No. 1, Section 4, Roosevelt Road, Taipei, 10617 Taiwan; 5 School of Medicine, National Taiwan University, Taipei, Taiwan; 6 National Health Insurance Administration, Ministry of Health and Welfare, Taipei, Taiwan; 7 Department of Medical Imaging, National Taiwan University Cancer Center, National Taiwan University College of Medicine, Taipei, Taiwan; 8 Department of Internal Medicine, Division of Gastroenterology and Hepatology, National Taiwan University Hospital, National Taiwan University College of Medicine, Taipei, Taiwan; 9 Internal Medicine, National Taiwan University College of Medicine, No. 7, Chung-Shan South Road, Taipei, 10002 Taiwan; 10 NVIDIA, Bethesda, MD 20814 USA

**Keywords:** Pancreatic cancer, Pancreatic ductal adenocarcinoma, Radiomics, Machine learning, Computer-aided detection

## Abstract

**Background:**

CT is the major detection tool for pancreatic cancer (PC). However, approximately 40% of PCs < 2 cm are missed on CT, underscoring a pressing need for tools to supplement radiologist interpretation.

**Methods:**

Contrast-enhanced CT studies of 546 patients with pancreatic adenocarcinoma diagnosed by histology/cytology between January 2005 and December 2019 and 733 CT studies of controls with normal pancreas obtained between the same period in a tertiary referral center were retrospectively collected for developing an automatic end-to-end computer-aided detection (CAD) tool for PC using two-dimensional (2D) and three-dimensional (3D) radiomic analysis with machine learning. The CAD tool was tested in a nationwide dataset comprising 1,477 CT studies (671 PCs, 806 controls) obtained from institutions throughout Taiwan.

**Results:**

The CAD tool achieved 0.918 (95% CI, 0.895–0.938) sensitivity and 0.822 (95% CI, 0.794–0.848) specificity in differentiating between studies with and without PC (area under curve 0.947, 95% CI, 0.936–0.958), with 0.707 (95% CI, 0.602–0.797) sensitivity for tumors < 2 cm. The positive and negative likelihood ratios of PC were 5.17 (95% CI, 4.45–6.01) and 0.10 (95% CI, 0.08–0.13), respectively. Where high specificity is needed, using 2D and 3D analyses in series yielded 0.952 (95% CI, 0.934–0.965) specificity with a sensitivity of 0.742 (95% CI, 0.707–0.775), whereas using 2D and 3D analyses in parallel to maximize sensitivity yielded 0.915 (95% CI, 0.891–0.935) sensitivity at a specificity of 0.791 (95% CI, 0.762–0.819).

**Conclusions:**

The high accuracy and robustness of the CAD tool supported its potential for enhancing the detection of PC.

**Supplementary Information:**

The online version contains supplementary material available at 10.1186/s12885-023-10536-8.

## Background

Pancreatic cancer (PC), the most lethal cancer, is projected to become the second leading cause of cancer deaths in the US by 2030 with a 5-year survival rate of about only 11% [1, 2]. Because patient survival rapidly diminishes with increasing tumor size [3], early detection is the most effective strategy to improve the grave prognosis [4]. Computed tomography (CT) is the major imaging modality for detecting PC, but early PCs are often obscure or even invisible to the naked eye on CT, with approximately 40% of PCs smaller than 2 cm being missed by radiologist interpretation [5]. Furthermore, the diagnostic performance of CT is interpreter-dependent and could be adversely affected by increasing radiologist workload [6]. Therefore, an effective tool that can assist radiologists in detecting PC is urgently needed and represents a major unmet clinical need.

Radiomics is a method which extracts quantitative information on density, shape, and texture from images for subsequent data mining [7]. Analysis of radiomic features with machine learning algorithms has shown great promise in medical image analysis [8]. In a recent proof of concept study, we identified distinguishing two-dimensional (2D) radiomic features of PC on CT and showed that patch-based 2D radiomic analysis with a machine learning model could distinguish CT studies of PC patients and controls with 95% accuracy in a local test dataset [8]. However, in that study the pancreas and tumor were manually labeled by radiologists for subsequent radiomic analysis, limiting its applicability in clinical settings. While the adoption of a patchwise 2D analytic approach in that study enabled detailed fine-grained assessment in each subregion of the pancreas, loss of information was inevitable compared with three-dimensional (3D) analytic approaches [9]. The generalizability of the trained model to external datasets also needs further validation. To be clinically applicable, a computer-aided detection (CAD) tool must achieve segmentation (i.e., identifying the pancreas and tumor) and classification (PC vs non-PC) with minimal human labor and deliver robust performance in real clinical settings.

Therefore, this study investigated 2D and 3D radiomic analysis for detecting PC on CT, with automatic segmentation of the pancreas and tumor by deep learning (DL). An end-to-end CAD tool based on 2D and 3D radiomic analysis combined was further developed and tested with prospectively collected CT images from real clinical practice throughout Taiwan to ascertain its generalizability.

## Methods

This retrospective study was conducted in accordance with the Declaration of Helsinki and approved by the Institute Research Ethical Committee of National Taiwan University Hospital (NTUH 201710050RINA, 201904116RINC), which waived the requirement for informed consent from individual patients.

### Local dataset and manual image segmentation

**Fig. 1:**
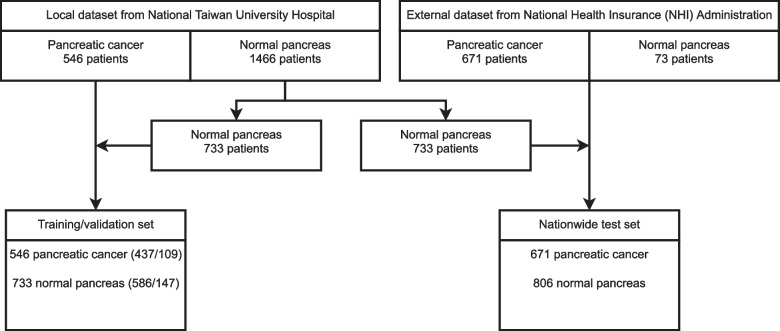
Flowchart of datasets

Patients with histologically- or cytologically-confirmed pancreatic adenocarcinoma were identified from the Cancer Registry of National Taiwan University Hospital (NTUH), a tertiary referral center with a large volume of PC. CT images of those PC patients and subjects with normal or unremarkable pancreas according to the formal radiologist reports were extracted from the imaging archive of NTUH for further review to construct the local datasets. If an individual patient underwent multiple CT examinations, only the one that immediately preceded the diagnosis of PC was used. In total, contrast-enhanced portal venous CT images of 546 confirmed PC patients between January 1, 2005, and December 31, 2019, and 1,466 subjects who underwent CT during the same period with a negative or unremarkable pancreas in the radiologist report were included in the local dataset. 733 controls were randomly selected from the local dataset to construct the nationwide test set (see below), whereas all cases and the remaining controls were randomly divided into a local training set (437 PCs, 586 controls) and a validation set (109 PCs, 147 controls) (Fig. 1).

CT examinations included in this study were performed with one of 47 scanners from six different manufacturers (GE Healthcare, Siemens Healthcare, Philips Healthcare, Toshiba, Picker International, and Hitachi Medical Corporation) with 100, 120, or 130 kV, automatic mA control, and without noise reduction. All CT images were obtained in the portal venous phase with intravenous administration of contrast medium (1.5 mL per kg body weight, with an upper limit of 150 mL). Each slice had a size of 512 × 512 pixels, with a thickness ranging between 0.7 mm and 1.5 mm that was reconstructed into 5-mm for subsequence analysis. The pancreas and tumor on the CT images of PC patients were manually segmented and labeled as regions of interest (ROIs) for model training by one of two experienced abdominal radiologists (P-TC and K-LL) who had over 8 years and 23 years of experience, respectively, with reference to the results of other examinations and surgical findings when needed.

### Population-based test set

Taiwan’s National Health Insurance (NHI) is a single-payer compulsory health insurance program that covers inpatient and outpatient care for 99.8% of the population and is contracted with 92.7% (21,463/23,164) of the institutions in Taiwan [10]. Up to July 2021, around 2.9 million imaging studies performed in daily clinical practice were uploaded by institutions throughout Taiwan to the Applications of Artificial Intelligence in Medical Images Database of NHI. Patients diagnosed with PC throughout Taiwan were identified by searching for recipients of the Severe Illness Certificate issued for the International Classification of Diseases—10th Revision-Clinical Modification (ICD-10-CM) [11] code of C25 (malignant neoplasm of the pancreas) and its sub-items in the NHI Major Illness/Injury Certificate database, and the Applications of Artificial Intelligence in Medical Images Database was searched to retrieve CT studies of those patients with PC. If multiple CT studies were identified for an individual patient, only the study closest to the date of certificate issuance was retrieved, yielding CT studies of 671 cases with newly confirmed PC diagnosis between January 1, 2018, and July 31, 2019, throughout Taiwan. For controls with normal pancreas, CT studies performed for pre-donation evaluation of all kidney donors and liver donors during the same period were retrieved from the Applications of Artificial Intelligence in Medical Images Database and reviewed by a radiologist (P-TC) to confirm the absence of radiological abnormalities in the pancreas, yielding 73 control subjects. To balance the number of cases and controls, the 733 controls randomly selected from the local dataset were combined with the controls from the NHI dataset to form the nationwide test set (671 PCs, 806 controls) (Fig. 1).

### Two- and three-dimensional radiomic analyses

The radiomics-based classification module consisted of three components: a 3D radiomics model (3D analysis), a 2D patch-based radiomics model (2D analysis), and a logistic regression model using the outputs of the former two radiomics models as independent variables (combined analysis) to predict the probability of PC. The 3D analysis examined the whole pancreas by 3D radiomic features, whereas in the 2D analysis the pancreas on each 2D slice was cropped into patches for subsequent extraction of 2D radiomic features.

For 3D analysis, the pancreas including the tumor (if present) was segmented using a previously trained automatic deep learning segmentation model [12]. This segmentation model was trained with CT images of 437 PC patients from the NTUH training and validation datasets and those of 393 subjects from three external datasets based on a model from coarse-to-fine network architecture search (C2FNAS) [13] (Additional file 1). For model training in the 2D analysis, segmentation of the pancreas and tumor was performed manually by radiologists in the images of PC patients and by the automatic segmentation model in the images of controls. During model testing, the automatic segmentation model was used for segmenting the pancreas and tumor in both 2D and 3D analyses.

All radiomic features in this study were extracted using an open-source platform (PyRadiomics) [14]. To eliminate bias resulting from the differences in spacing when extracting the radiomic features, all the images and segmentation labels were resampled to the spacing of 1 × 1 × 5 mm using linear interpolation and nearest-neighbor interpolation, respectively. The bin width for computing texture features was fixed at 16.

### Training of classification models based on three-dimensional radiomic features

For 3D analysis, a 3D radiomics model was trained from the NTUH training set. The union of the pancreas and tumor, segmented by the automatic segmentation model, in the 3D CT volume served as the volume of interest (VOI) for subsequent extraction of 3D radiomic features. A total of 1183 features, including first-order and higher-order texture features on original and filtered images, were extracted from the VOI (Additional file 1). To differentiate whether a pancreas included a tumor based on 3D radiomic features, the 1183 features of all data from the training set were inputted into XGBoost, a widely used machine learning algorithm based on gradient boosting decision tree [15], to train a classification model. To mitigate overfitting, the training process was terminated when the area under the receiver operating characteristic curve (AUC) on the validation set was not increased for 30 iterations, and the model in the training process that had the highest AUC on the validation set was selected as the final model for 3D analysis. The loss function of this XGBoost model was set as logistic loss and the resultant probability served as the output of the 3D analysis.

### Training of classification models based on two-dimensional patch-based radiomic features

In 2D analysis, every patch generated from the ROI (i.e. pancreas and tumor) in a CT study was analyzed by a 2D patch-based radiomics model to predict the probability of cancer in each patch, and the patient was predicted as with or without PC by jointly considering the predicted probabilities of all patches of the patient. The union of pancreas and tumor on the images was set as the ROI. After resampling, the images were cropped into 20 × 20 pixel square subregions (i.e. patches) on the axial (x–y) plane, using a moving window with a stride of five pixels. The patches which had more than 5% of the area overlapping with ROI were treated as valid patches and included in the analysis. The patches containing any portion of the tumor were labeled as cancerous patches, whereas the patches containing no tumor were labeled as non-cancerous. A total of 545 features were extracted from the ROI in each patch (Additional file 1). All the features of patches in the training set were then input into XGBoost with the logistic loss function for distinguishing cancerous patches from non-cancerous patches.

To determine whether a patient had a tumor, a heatmap was generated for each patient by aggregating the prediction from the 2D patch-based radiomic model. More specifically, all valid patches extracted from the ROI of a specific patient were input into the trained XGBoost model to obtain the probabilities of having a tumor, and then the prediction results of all patches were assembled into a heatmap. The value of each pixel in the heatmap was defined as the average of the predicted probabilities of the patches that contained this pixel. Then the area of the high-risk region of a CT study was defined as the area of the largest region formed by contiguously neighboring high-risk pixels among all the axial planes, with the threshold for classifying a pixel as high-risk set at the value corresponding to the highest AUC in differentiating between PCs and controls in the validation set, searched from 0.05 to 0.95 with a step of 0.01. The area of the high-risk region was used as the output of the 2D analysis.

### Development of CAD tool based on automatic segmentation and two- and three-dimensional radiomic analysis combined

**Fig. 2:**
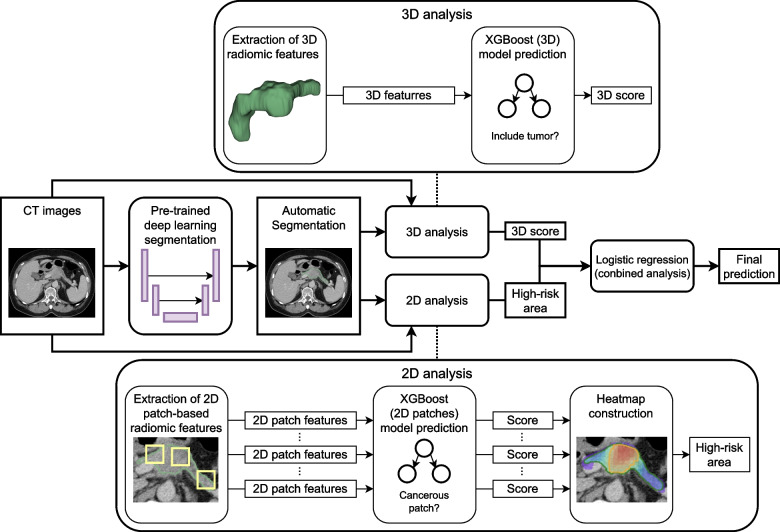
Workflow of developed CAD tool

An automatic CAD tool for PC comprising a DL-based segmentation module and a radiomics-based classification module was developed (Fig. 2). The pancreas and tumor (if present) on CT images were first automatically segmented by the previously trained DL segmentation model [12] and then subject to the extraction of 3D and 2D radiomic features, which were subsequently analyzed by the trained 3D and 2D radiomic analysis models, respectively. The resultant probability of PC generated from the 3D analysis and the area of the high-risk region from the 2D analysis were then input into a logistic regression model trained with the validation set to yield the final prediction regarding whether the CT study harbored PC (combined analysis).

### Model testing and statistical analysis

The performance of the automatic CAD workflow with 3D analysis, 2D analysis, and combined analysis was tested with the nationwide test set. The automatic segmentation was used as both VOI in 3D analysis and ROI in 2D analysis. For differentiating whether a patient had PC for each analysis, the cutoff was selected as the threshold that corresponded to the point with the highest Youden index (i.e. sensitivity + specificity—1) on the ROC curve plotted by the outputs of the corresponding analysis from all patients in the validation set. Model performance was assessed with ROC curves and associated AUCs. Sensitivity, specificity, and accuracy were ascertained with the respective exact confidence intervals (CI) calculated based on binomial distributions. For comparison between groups, Fisher exact test and Mann–Whitney U test were used for categorical variables and continuous variables, respectively. Comparison of AUCs between various analyses was conducted using the pROC package in R [16, 17] with paired Delong’s method.

## Results

### Training and testing of three-dimensional radiomics model

**Fig. 3:**
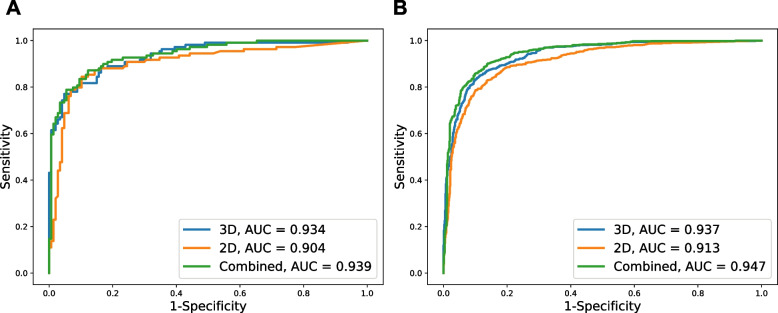
Receiver operating characteristic curves. **A**: local validation set, **B**: nationwide test set

The clinical characteristics of the subjects in the local dataset are summarized in Table 1. In the validation set, 3D radiomic analysis with trained model achieved 0.771 (0.680–0.846) sensitivity, 0.952 (0.904–0.981) specificity, and 0.875 (0.828–0.913) accuracy [AUC 0.934 (0.905–0.964)] (Table 2, Fig. 3A). In the nationwide test set, the 3D analysis with trained model achieved 0.796 (0.763–0.826) sensitivity, 0.920 (0.900–0.938) specificity, and 0.864 (0.845–0.881) accuracy [AUC 0.937 (0.925–0.949)]. The specificity in the controls from NHI [0.944 (0.864–0.985)] was comparable with that in the controls from NTUH [0.918 (0.896–0.937)] (Table 2, Fig. 3B).

**Table 1:** Characteristics of patients in local training/validation datasets

	PCs (*n* = 546)	Control (*n* = 733)
Age (year), mean ± SD	64.6 ± 11.8	54.2 ± 16.2
Male, n (%)	297 (54.4%)	374 (51.0%)
Stage I/II/III/IV (%)	4.9/40.5/14.5/40.1	-
Tumor size < 2/2–4/ > 4 cm (%)	24.7/49.5/25.8	-

*SD* standard deviation

**Table 2:** Performance of 3D analysis, 2D analysis, and combined analysis in various sets

	Sensitivity	Specificity	Accuracy	AUC	LR +	LR-
Local validation set (109 PCs / 147 controls)						
3D analysis	0.771 (0.680–0.846)	0.952 (0.904–0.981)	0.875 (0.828–0.913)	0.934 (0.905–0.964)	16.18 (7.80–33.59)	0.24 (0.17–0.34)
2D analysis	0.844 (0.762–0.906)	0.898 (0.837–0.942)	0.875 (0.828–0.913)	0.904 (0.863–0.946)	8.27 (5.09–13.45)	0.17 (0.11–0.27)
Combined analysis	0.872 (0.794–0.928)	0.878 (0.813–0.926)	0.875 (0.828–0.913)	0.939 (0.911–0.968)	7.12 (4.59–11.04)	0.15 (0.09–0.24)
3D and 2D in series	0.734 (0.641–0.814)	0.980 (0.942–0.996)	0.875 (0.828–0.913)	-	35.96 (11.67–110.85)	0.27 (0.20–0.37)
3D and 2D in parallel	0.881 (0.805–0.935)	0.871 (0.806–0.920)	0.875 (0.828–0.913)	-	6.81 (4.45–10.43)	0.14 (0.08–0.23)
Nationwide test set (671 PCs / 805 controls)^a^						
3D analysis	0.796 (0.763–0.826)	0.920 (0.900–0.938)	0.864 (0.845–0.881)	0.937 (0.925–0.949)	10.01 (7.89–12.70)	0.22 (0.19–0.26)
2D analysis	0.861 (0.833–0.887)	0.822 (0.794–0.848)	0.840 (0.820–0.858)	0.913 (0.898–0.927)	4.85 (4.17–5.64)	0.17 (0.14–0.20)
Combined analysis	0.918 (0.895–0.938)	0.822 (0.794–0.848)	0.866 (0.847–0.883)	0.947 (0.936–0.958)	5.17 (4.45–6.01)	0.10 (0.08–0.13)
3D and 2D in series	0.742 (0.707–0.775)	0.952 (0.934–0.965)	0.856 (0.837–0.874)	-	15.32 (11.24–20.87)	0.27 (0.24–0.31)
3D and 2D in parallel	0.915 (0.891–0.935)	0.791 (0.762–0.819)	0.848 (0.828–0.866)	-	4.39 (3.83–5.03)	0.11 (0.08–0.14)

^a^One control was excluded due to the failure of automatic segmentation

*LR +* positive likelihood ratio, *LR- *negative likelihood ratio

**Fig. 4:**
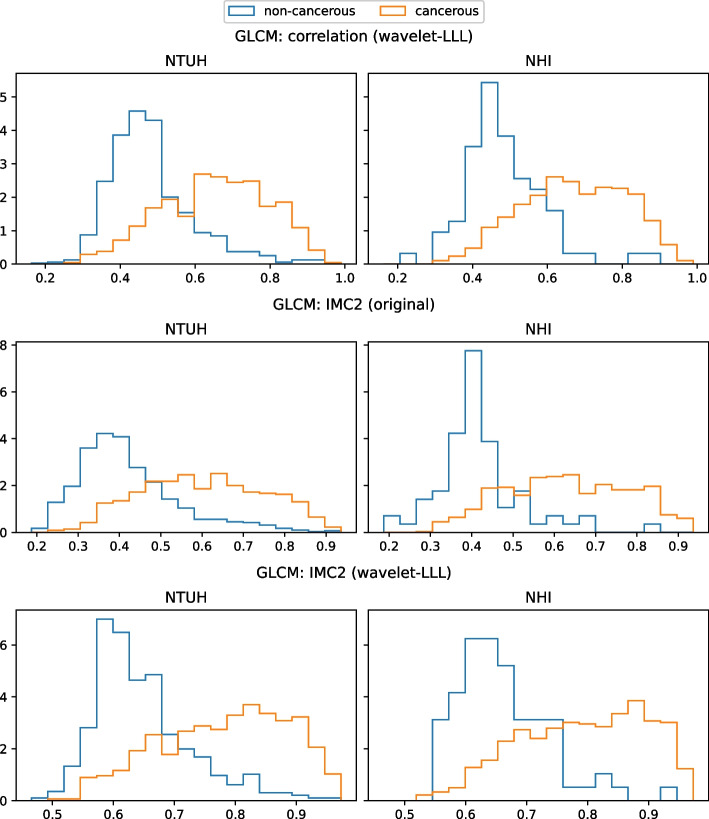
Density histograms of the top three important features in the 3D radiomics XGBoost model

Among the 1183 radiomic features, the *p*-values of 236 (19.9%) features were smaller than 0.001. The top 10 differential features (i.e., features with the highest gain values) according to the 3D radiomics XGBoost model are summarized in Table 3. The top three differential features were GLCM: correlation (wavelet-LLL), GLCM: IMC2 (original), and GLCM: IMC2 (wavelet-LLL), all of which are generally categorized as texture features as they measure the correlation between the gray level of a voxel and the gray levels of its surrounding voxels in the VOI. Values of these 3 texture features were consistently higher in PCs compared with those in controls in both the local dataset and the nationwide dataset (Fig. 4).

**Table 3:** Top 10 radiomic features with highest gain value in 3D-analysis patch-based model

Feature	Gain value
GLCM: correlation (wavelet-LLL)	54.27
GLCM: IMC2 (original)	36.62
GLCM: IMC2 (wavelet-LLL)	24.27
First order: mean (log-sigma-3–0-mm-3D)	17.12
GLDM: small dependence emphasis (wavelet-HHH)	13.34
GLDM: gray level variance (wavelet-HHH)	11.97
First order: minimum (wavelet-LLL)	11.80
GLCM: inverse variance (wavelet-LHH)	10.43
GLCM: IMC2 (wavelet-HHL)	10.14
GLCM: inverse variance (wavelet-HHH)	9.67

*GLCM* Gray Level Co-occurrence Matrix, *IMC* Informational Measure of Correlation, *GLDM*: Gray Level Dependence Matrix

### Training and testing of two-dimensional radiomics model

**Fig. 5:**
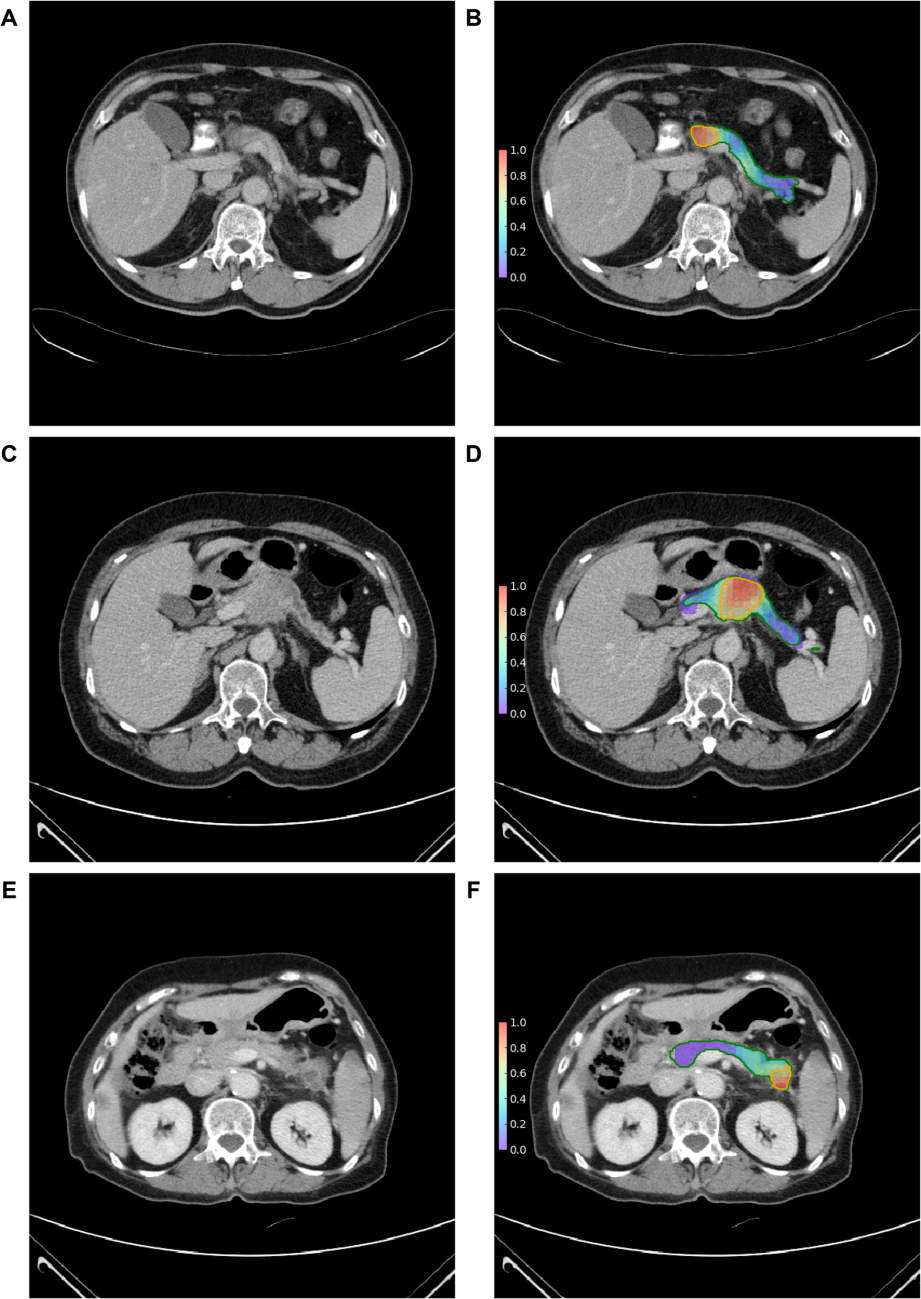
Original CT images and corresponding heatmaps. Original CT images (**A**, **C**, **E**) and corresponding heatmaps (**B**, **D**, **F**) show the patch-wise probability of cancer according to 2D radiomic analysis. The regions encircled by green lines are the pancreas with/without tumor segmented by the deep learning segmentation model. The regions encircled by orange lines indicate areas with a high probability of cancer

**Fig. 6:**
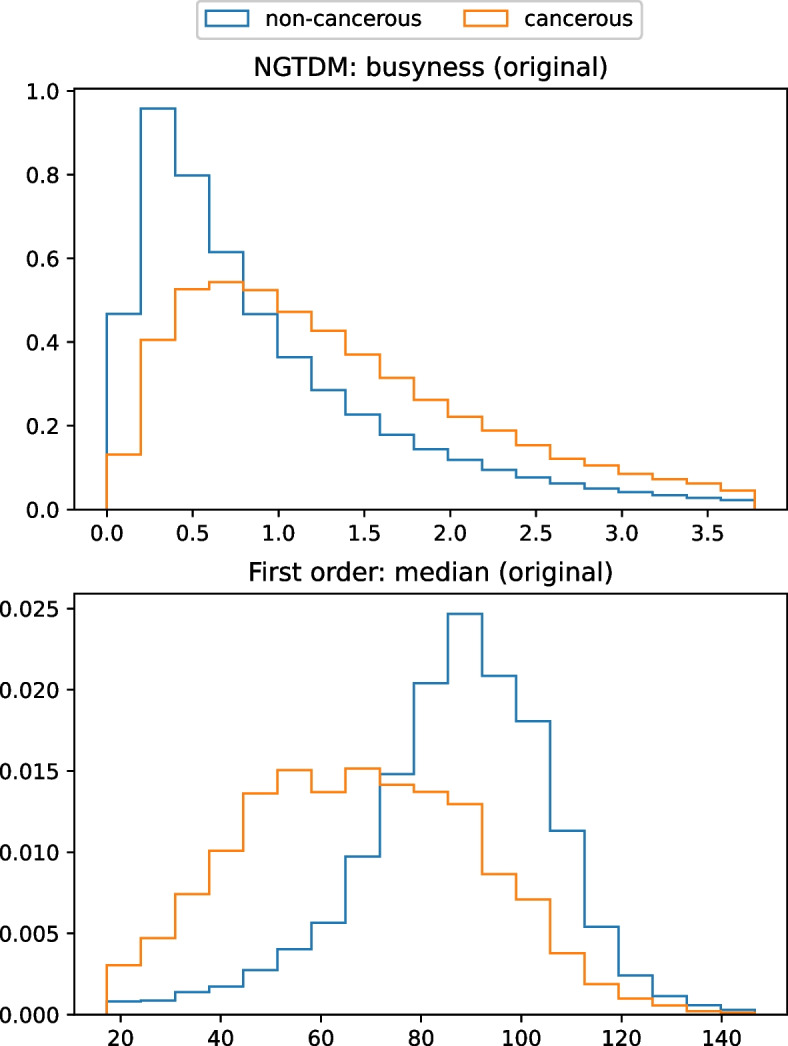
Density histograms of the top two important features in the 2D patch-based radiomics XGBoost model

360,379 cancerous patches and 80,114 non-cancerous patches were generated to train the XGBoost model which classified patches based on 2D radiomic features (i.e., patched-based analysis). In the validation set, the trained patch-based model achieved 0.826 (0.820–0.832) sensitivity, 0.888 (0.885–0.890) specificity, and 0.877 (0.874–0.879) accuracy [AUC 0.934 (0.932–0.936)]. Figure 5 illustrates heatmaps showing the patch-wise probability of PC predicted by the trained patch-based model. Among the 546 radiomic features, the *p*-values of 534 (97.8%) features were smaller than 0.001. The top 10 differential features according to the patch-based model are summarized in Table 4. The top-ranking feature of NGTDM: business (original) is a texture feature that measures the heterogeneity inside the ROI, and its values were higher in cancerous patches compared with noncancerous patches in the local dataset (Fig. 6), indicating that PCs were more heterogeneous with a coarser texture. The second-ranked feature First order: median (original) measures the median intensity inside the ROI, and its values were lower in cancerous patches compared with noncancerous patches in the local dataset (Fig. 6). Features values in the nationwide dataset could not be analyzed because of the regulations of NHI. Differentiating between studies of patients with and without PC (ie., patient-based analysis) based on the heatmaps generated by the patch-based model achieved 0.844 (0.762–0.906) sensitivity, 0.898 (0.837–0.942) specificity, and 0.875 (0.828–0.913) accuracy in the validation set [AUC 0.904 (0.863–0.946)] (Table 2, Fig. 3A).

**Table 4:** Top 10 radiomic features with highest gain value in 2D-analysis patch-based model

Feature	Gain value
NGTDM: busyness (original)	9735.6
First order: median (original)	8999.0
GLSZM: large area emphasis (wavelet-HL)	4841.2
GLDM: dependence entropy (wavelet-LL)	4013.8
First order: mean absolute deviation (wavelet-HL)	3577.7
NGTDM: coarseness (wavelet-LH)	2750.1
GLDM: dependence non-uniformity (wavelet-HL)	2456.0
GLRLM: long run emphasis (wavelet-HL)	2421.2
GLCM: correlation (wavelet-LL)	2407.8
First order: skewness (original)	2298.6

*NGTDM* Neighboring Gray Tone Difference Matrix, *GLSZM* Gray Level Size Zone Matrix, *GLDM* Gray Level Dependence Matrix, *GLRLM* Gray Level Run Length Matrix, *GLCM* Gray Level Co-occurrence Matrix

In the nationwide test set, patient-based analysis based on the trained 2D patched-based yielded 0.861 (0.833–0.887) sensitivity, 0.822 (0.794–0.848) specificity, and 0.840 (0.820–0.858) accuracy, with a slightly lower AUC [0.913 (0.898–0.927)] compared with that achieved with 3D analysis (*p* = 0.001). The specificity in the controls from NHI [0.861 (0.759–0.931)] was comparable with that in the controls from NTUH [0.819 (0.789–0.846)] (Table 2, Fig. 3B).

### CAD tool for pancreatic cancer detection based on two- and three-dimensional radiomic analysis combined

The trained logistic regression model for generating the final prediction of the probability of PC in the CAD tool was *logit*(*p*) = 3.95759716 × (resultant probability of PC from 3D analysis) + 0.00241766494 × [area of the high-risk region from 2D analysis (mm^2^)]—2.79234888, where *p* represented the probability of the CT study harboring PC. The CAD tool achieved 0.872 (0.794–0.928) sensitivity, 0.878 (0.813–0.926) specificity, and 0.875 (0.828–0.913) accuracy in the validation set [AUC 0.939 (0.911–0.968)] (Table 2, Fig. 3A).

In the nationwide test set, the CAD tool achieved 0.918 (0.895–0.938) sensitivity, 0.822 (0.794–0.848) specificity, and 0.866 (0.847–0.883) accuracy, with a significantly higher AUC [0.947 (0.936–0.958)] compared with that of 2D analysis (*p* < 0.001) and 3D analysis (*p* = 0.004), respectively. The specificity in the controls from NHI [0.792 (0.680–0.878)] was comparable with that in the controls from NTUH [0.825 (0.796–0.852)] (Table 2, Fig. 3B).

### Sensitivity according to tumor size

The sensitivity for PC stratified by tumor size is summarized in Table 5. A positive association between sensitivity and tumor size was noted with either 2D or 3D analysis and combined analysis (all Ptrend < 0.001). For tumors < 2 cm, the CAD tool achieved significantly higher sensitivity [0.707 (0.602–0.797)] compared with 3D analysis [0.522 (0.415–0.627), *p* = 0.015] and 2D analysis [0.467 (0.363–0.574), *p* = 0.002] in the nationwide test set. The sensitivity of the CAD tool for tumors 2–4 cm and larger than 4 cm was 0.934 (0.904–0.957) and 0.985 (0.957–0.997), respectively.

**Table 5:** Sensitivity of 3D analysis, 2D analysis, and combined analysis in various test sets stratified by tumor size

	Local validation set (*n* = 109)	Nationwide test set (*n* = 671)
< 2 cm		
3D analysis	12/25, 0.480 (0.278–0.687)	48/92, 0.522 (0.415–0.627)
2D analysis	16/25, 0.640 (0.425–0.820)	43/92, 0.467 (0.363–0.574)
Combined analysis	16/25, 0.640 (0.425–0.820)	65/92, 0.707 (0.602–0.797)^a^
2–4 cm		
3D analysis	47/59, 0.797 (0.672–0.890)	301/380, 0.792 (0.748–0.832)
2D analysis	51/59, 0.864 (0.750–0.940)	341/380, 0.897 (0.862–0.926)^b^
Combined analysis	54/59, 0.915 (0.813–0.972)	355/380, 0.934 (0.904–0.957)^c^
> 4 cm		
3D analysis	25/25, 1.000 (0.863–1.000)	185/199, 0.930 (0.885–0.961)
2D analysis	25/25, 1.000 (0.863–1.000)	194/199, 0.975 (0.942–0.992)
Combined analysis	25/25, 1.000 (0.863–1.000)	196/199, 0.985 (0.957–0.997)^d^

^a^ *p* = 0.015 compared with 3D, *p* = 0.002 compared with 2D

^b^ *p* < 0.001 compared with 3D

^c^ *p* < 0.001 compared with 3D

^d^ *p* = 0.011 compared with 3D

### Likelihood ratios of pancreatic cancer

In the nationwide test set, the positive likelihood ratios (+ LR) and negative likelihood ratios (-LR) of the CAD tool were 5.17 (4.45–6.01) and 0.10 (0.08–0.13), respectively (Table 2). When both 3D and 2D analyses predicted a study as positive, the + LR of PC increased to 15.32 (11.24—20.87). When both 3D and 2D analyses predicted a study as negative, the -LR of PC was 0.11 (0.08—0.14) (Table 6). For clinical settings where high specificity is needed, using 2D and 3D analyses in series (i.e., the study predicted as positive if both tests were positive) yielded 0.952 (0.934–0.965) specificity with a sensitivity of 0.742 (0.707–0.775). When high sensitivity is needed, using 2D and 3D analyses in parallel (i.e., the study predicted as positive if either test was positive) yielded 0.915 (0.891–0.935) sensitivity at a specificity of 0.791 (0.762–0.819) (Table 2).

**Table 6:** Likelihood ratios provided by the CAD tool

	3D + , 2D +	3D + , 2D-	3D-, 2D +	3D-, 2D-
Local validation set				
N (%) of PC	80 (73.4%)	4 (3.7%)	12 (11.0%)	13 (11.9%)
N (%) of control	3 (2.0%)	4 (2.7%)	12 (8.2%)	128 (87.1%)
Likelihood ratio	35.96 (11.67—110.85)	1.35 (0.35—5.27)	1.35 (0.63—2.89)	0.14 (0.08—0.23)
Nationwide test set				
N (%) of PC	498 (74.2%)	36 (5.4%)	80 (11.9%)	57 (8.5%)
N (%) of control	39 (4.8%)	25 (3.1%)	104 (12.9%)	637 (79.1%)
Likelihood ratio	15.32 (11.24—20.87)	1.73 (1.05—2.85)	0.92 (0.70—1.21)	0.11 (0.08—0.14)

## Discussion

This study combined a segmentation DL model with 2D and 3D radiomic analysis for detecting PC on contrast-enhanced CT images. We further developed an automatic end-to-end CAD tool which combined 2D and 3D radiomic analysis and did not require manual image preprocessing and labeling/segmentation. In a test set comprising real-world data prospectively collected from institutions across Taiwan, the CAD tool achieved 0.918 sensitivity and 0.822 specificity, confirming its robustness and generalizability.

We have previously identified novel distinguishing CT radiomic features of PC and showed that patch-based 2D radiomic analysis with a machine learning model accurately detected PC in an independent local dataset [18]. Another previous study also employed 3D radiomic analysis of the pancreas for distinguishing PC on CT [19] and achieved 0.992 accuracy in a test dataset from the same institution. However, in that study, the generalizability of the trained model was not assessed with external images and the VOIs (ie., pancreas and tumor) were manually labeled by radiologists. By contrast, in this study the ROIs and VOIs were automatically segmented by our DL segmentation model for subsequent radiomic analysis, and accurate detection of PC was achieved through the combination of patch-wise 2D and volumetric 3D radiomic analysis. In a real-world dataset prospectively collected from institutions throughout Taiwan, our end-to-end CAD tool achieved 0.918 sensitivity (0.707 for PCs < 2 cm) and 0.822 specificity, providing strong support for its robustness and generalizability.

This study is the first to conduct an in-depth comparative analysis between adopting 2D versus 3D analytic approach for radiomic analysis of the pancreas. The findings of this study demonstrated that while 3D radiomic analysis outperformed 2D radiomic analysis for detecting PC, combining both analyses further improved the performance compared with 3D analysis alone. The major advantage of the 2D analysis was that the pancreas was cropped into overlapping patches that are subject to radiomic analysis individually. Therefore, each fine-grained subregion was subject to multiple rounds of radiomic analysis, each round analyzed with different neighboring subregions, and thereby might increase the sensitivity for detection [20]. However, the 2D analytic approach could not account for the correlations between foci that are adjacent to each other in 3-D space but separated into different 2D slices. By contrast, 3D radiomic analysis could capture and take into consideration the correlations between neighboring foci in differentiating between cancerous and noncancerous pancreas. Notably, combining 2D and 3D analysis significantly improved the sensitivity for PC < 2 cm compared with either analysis alone (combined: 0.707, 2D: 0.467, 3D: 0.522), and previous research showed that approximately 40% of PCs smaller than 2 cm were missed on CT by human interpretation [5].

This study provides novel insights into the differential radiomic characteristics of PC. While the identified differential 3D radiomic features are either high order features or derived from filtered images and thus difficult to interpret, the results of 3D radiomic analysis indicated that differences in texture most distinctly differentiate between PC and noncancerous pancreas. In contrast, the finding of increased NGTDM: busyness (original), a measure of heterogeneity, and decreased First order: median (original), a measure of intensity, as the major differential 2D radiomic features of PC corresponded with the typical manifestations of PCs as heterogeneous hypodense masses on CT [21, 22] and abrogated our previous study which first reported these two features as key differential features of PC [18]. Further research to explore the potential correlations between these features and clinicopathological characteristics or treatment response is warranted.

Besides making a binary prediction (PC vs non-PC), the CAD tool could further provide LRs to better assist clinicians in determining the subsequent diagnostic-therapeutic process. Endoscopic ultrasound-guided tissue sampling enables preoperative differentiation between PC and various noncancerous mimickers to avoid unnecessary surgery but carries a risk of tumor dissemination and false negativity [23]. The general consensus is that patients with resectable pancreatic masses can undergo surgery without preoperative tissue sampling if PC is highly favored based on imaging and clinical grounds, and the need for tissue sampling should be carefully weighed considering the likelihood of PC vs alternative diagnoses, surgical candidacy, and risk of biopsy-related tumor dissemination [24, 25]. LR is a quantitative measure of the confidence of the binary prediction and can be multiplied with the pre-test odds determined based on clinical grounds and clinical experience to derive the post-test odds and probability of PC. In addition to the LRs based on the logistic regression model combining both 2D and 3D analyses, the CAD tool can also provide the LR based on the individual results of 2D and 3D analyses, thereby better informing the clinicians in choosing between direct surgery and tissue sampling. When maximal sensitivity or specificity is needed given the clinical scenario, the final prediction of the CAD tool could also be based on using 2D and 3D analysis in parallel or in series. Moreover, the pancreatic lesion identified by the segmentation model can serve to indicate the possible location of the tumor for further review by radiologists.

This study had several strengths. By integrating a DL segmentation model trained with images from multiple institutions and races/ethnicities [12] and radiomic analysis with machine learning, the resultant CAD tool enables automatic end-to-end analysis without requiring manual image annotation/processing. The novel approach of combining 2D and 3D radiomic analysis which respectively provides patch-wise and global interrogation of the pancreas and are thus complementary yielded better performance compared with either analysis alone. Furthermore, the prospectively collected real-world population-based test set included variations in imaging equipment/parameter and quality inherent in real clinical practice and thus was the most rigorous test set ever used in studies on the usefulness of CT radiomics in detecting PC. The ability to achieve high accuracy in such a test set provided strong support for the robustness and generalizability of the CAD tool. Given that the diagnostic performance of radiologists is adversely affected by overloading and disparities in expertise/experience in the actual clinical environment, this tool holds potential for supplementing radiologists to reduce miss rate and enhance the early detection of PC.

This study also had limitations. Radiologist reports were not available from the NHI dataset; therefore, we could not compare the performance of the CAD tool with that of radiologist interpretation. Secondly, the Taiwanese population from which the nationwide test set was derived is predominantly Asian. While this study attested to the generalizability of the CAD tool in the Taiwanese and perhaps Asian populations, the potential generalizability to other races and ethnicities requires further evaluation. Thirdly, this study focused on ascertaining the robustness of radiomics in differentiating between cases with PC and controls with normal pancreas in real-world multi-institutional settings. Whether radiomics can differentiate between PC and non-PC pancreatic diseases needs to be investigated in future research.

## Conclusions

In conclusion, this study developed an end-to-end CAD tool for PC based on 2D and 3D radiomic analysis with machine learning. The CAD tool accurately and robustly detected PC on contrast-enhanced CT images and thus could be used to enhance the detection of PC.

## Supplementary Information

**Additional file 1. **Supplemental information on training of the automatic segmentaion model and extraction of radiomic features.

## Data Availability

The availability of the dataset from National Taiwan University Hospital is subject to the regulations and policies of the hospital and thus not publicly available. Data from National Health Insurance Medical Images Database were provided to this study by National Health Insurance Administration of Taiwan, and the availability of the dataset is subject to the regulations and decisions of the Administration.

## Abbreviations

PC: Pancreatic cancer
CT: Computed tomography
2D: Two-dimensional
3D: Three-dimensional
CAD: Computer-aided detection
DL: Deep learning
NTUH: National Taiwan University Hospital
ROI: Regions of interest
NHI: National Health Insurance
ICD-10-CM: International Classification of Diseases—10th Revision-Clinical Modification
C2FNAS: Coarse-to-fine network architecture search
VOI: Volume of interest
AUC: Area under the receiver operating characteristic curve
CI: Confidence intervals
+ LR: Positive likelihood ratios
-LR: Negative likelihood ratios
