# Supplementary material for: Detection of pancreatic cancer with two- and three-dimensional radiomic analysis in a nationwide population-based real-world dataset
Source: BMC Cancer. 2023 Jan 17;23:58. doi: 10.1186/s12885-023-10536-8 (PMC9843893; doi:10.1186/s12885-023-10536-8)
Supplement: Supplementary file 1 — Additional file 1. Supplemental information on training of the automatic segmentaion model and extraction of radiomic features. [file 12885_2023_10536_MOESM1_ESM.pdf]

# Supplemental Methods

## Training of deep learning segmentation model

To enable automatic segmentation of the pancreas including the tumor (if present) in CT images, a deep learning model was trained using 437 PDAC patients from the NTUH training and validation set and three external datasets, including the Medical Segmentation Decathlon Dataset (MSDD) from Memorial Sloan Kettering (MSK) Cancer Center comprising CT images with labeled pancreas and lesion segmentation of 281 patients with PDAC, intraductal papillary mucinous neoplasm (IPMN), or pancreatic neuroendocrine tumor (PNET) [1, 2], the Cancer Imaging Archive (TCIA) dataset comprising CT images of 82 subjects with labeled pancreas segmentation from the National Institutes of Health (NIH) Clinical Center [3–5], and the Synapse dataset from Vanderbilt University Medical Center containing CT scans of 30 subjects with labeled pancreas segmentation [6–8].

The resultant model from a coarse-to-fine network architecture search (C2FNAS) [9] was implemented and trained to automatically segment the pancreas along with the tumor. The manual segmentation of the pancreas and tumor served as the ROIs for training the segmentation model. For the data preprocessing, the CT volumes were transformed into R-A-S+ orientation, followed by resampling to 1 × 1 × 1 mm and intensity clipping to -200 to 250 Hounsfield units (HU). Finally, the intensity values were rescaled to [-1, 1]. Four NVIDIA GPUs (Quadro RTX8000) were used for the model training with batch size 8 and input cropping size [96, 96, 96]. The Adam optimizer was used for the training process with the loss function set as the summation of the Dice loss and the cross-entropy. The learning rates were scheduled by cosine annealing from $10^{-3}$ to $10^{-5}$.

**Extraction of radiomic features**

All radiomic features in this study were extracted by an open-source platform (PyRadiomics) [10]. Filters were applied on a given image before the feature extraction to increase the number of extracted features. More specifically, for extracting features from an image with corresponding ROI, each filter was applied on the original image to get filtered images. Then 91 features were extracted from the original image and each of the filtered images. Finally, all the features from different filtering of images were collected and seen as the radiomic features extracted from the target image. Suppose there are N filters to be applied, a total of (N+1) × 91 features were extracted. Because of the differences between 2D and 3D analytic approaches, the numbers of filters used during feature extraction were different between 2D and 3D analyses. Distinct features with similar purposes were preserved during the feature extraction process to increase the odds of capturing the subtle differential features of PC.

For extracting 3D radiomic features, the following 12 filters were used: 3 from Laplacian of Gaussian (LoG) filter (with the parameter sigma setting as 1, 2, or 3), 8 from wavelet transformation (with high pass filter [H] and low pass filter [L] as HHH, HHL, HLH, HLL, LHH, LHL, LLH, LLL), and 1 from the gradient filter. Therefore, a total of (12+1) × 91 = 1183 features were used for establishing 3D analysis.

For extracting 2D radiomic features, the following 5 filters were used: 4 from wavelet transformation (with high/low pass filters as HH, HL, LH, LL) and 1 from the gradient filter. A total of (5+1) × 91 = 546 features were extracted. However, due to the computational issue, the feature GLCM: IMC2 extracted from the gradient filter was excluded from the analysis. Therefore, a total of 545 features were used for establishing 2D analysis.

The 91 features that were extracted from the original image and filtered images were listed as follows:

- First order features: 10 percentile, 90 percentile, energy, entropy, interquartile range, kurtosis, maximum, mean, mean absolute deviation, median, minimum, range, robust mean absolute deviation, root mean squared, skewness, total energy, uniformity, variance.
- Gray Level Co-occurrence Matrix (GLCM) features: autocorrelation, cluster prominence, cluster shade, cluster tendency, contrast, correlation, difference average, difference entropy, difference variance, ID, IDM, IDMN, IDN, IMC1, IMC2, inverse variance, joint average, joint energy, joint entropy, maximum probability, sum entropy, sum squares.
- Gray Level Dependence Matrix (GLDM) features: dependence entropy, dependence non-uniformity, dependence non-uniformity normalized, dependence variance, gray-level non-uniformity, gray-level variance, high gray-level emphasis, large dependence emphasis, large dependence high gray-level emphasis, large dependence low gray-level emphasis, low gray-level emphasis, small dependence emphasis, small dependence high gray-level emphasis, small dependence low gray-level emphasis.
- Gray Level Run Length Matrix (GLRLM) features: gray-level non-uniformity, gray-level non-uniformity normalized, gray-level variance, high gray-level run emphasis, long-run emphasis, long-run high gray-level emphasis, long-run low gray-level emphasis, low gray-level run emphasis, run entropy, run-length non-uniformity, run-length non-uniformity normalized, run percentage, run variance, short-run emphasis, short-run high gray-level emphasis, short-run low gray-level emphasis.

- Gray Level Size Zone Matrix (GLSZM) features: gray-level non-uniformity, gray-level non-uniformity normalized, gray-level variance, high gray-level zone emphasis, large area emphasis, large area high gray-level emphasis, large area low gray-level emphasis, low gray-level zone emphasis, size zone non-uniformity, size zone non-uniformity normalized, small area emphasis, small area high gray-level emphasis, small area low gray-level emphasis, zone entropy, zone percentage, zone variance.
- Neighboring Gray Tone Difference Matrix (NGTDM) features: busyness, coarseness, complexity, contrast, strength.

## References

1. Medical Segmentation Decathlon. http://medicaldecathlon.com/index.html. Accessed 13 Oct 2020.

2. Simpson AL, Antonelli M, Bakas S, Bilello M, Farahani K, van Ginneken B, et al. A large annotated medical image dataset for the development and evaluation of segmentation algorithms. arXiv [cs.CV]. 2019.

3. Clark K, Vendt B, Smith K, Freymann J, Kirby J, Koppel P, et al. The Cancer Imaging Archive (TCIA): maintaining and operating a public information repository. J Digit Imaging. 2013;26:1045–57.

4. Pancreas-CT - The Cancer Imaging Archive (TCIA) Public Access - Cancer Imaging Archive Wiki. https://wiki.cancerimagingarchive.net/display/Public/Pancreas-CT. Accessed 13 Oct 2020.

5. Roth HR, Lu L, Farag A, Shin H-C, Liu J, Turkbey EB, et al. DeepOrgan: Multi-level Deep Convolutional Networks for Automated Pancreas Segmentation. In: Medical Image Computing and Computer-Assisted Intervention -- MICCAI 2015. Springer International Publishing; 2015. p. 556–64.

6. Xu Z, Lee CP, Heinrich MP, Modat M, Rueckert D, Ourselin S, et al. Evaluation of Six Registration Methods for the Human Abdomen on Clinically Acquired CT. IEEE Trans Biomed Eng. 2016;63:1563–72.

7. Bionetworks S. Synapse. 2019.

8. Gibson E, Giganti F, Hu Y, Bonmati E, Bandula S, Gurusamy K, et al. Multi-Organ

Abdominal Ct Reference Standard Segmentations. 2018.

9. Yu Q, Yang D, Roth H, Bai Y, Zhang Y, Yuille AL, et al. C2FNAS: Coarse-to-Fine Neural Architecture Search for 3D Medical Image Segmentation. In: Proceedings of the IEEE/CVF Conference on Computer Vision and Pattern Recognition. 2020. p. 4126–35.

10. van Griethuysen JJM, Fedorov A, Parmar C, Hosny A, Aucoin N, Narayan V, et al. Computational Radiomics System to Decode the Radiographic Phenotype. Cancer Res. 2017;77:e104–7.
